# The acute effect of beta-guanidinopropionic acid versus creatine or placebo in healthy men (ABC Trial): study protocol for a randomized controlled trial

**DOI:** 10.1186/s13063-015-0581-9

**Published:** 2015-02-22

**Authors:** Fares A Karamat, Deborah L Horjus, Yentl C Haan, Lisa van der Woude, Inge Oudman, Gert A van Montfrans, Joseph F Clark, Lizzy M Brewster

**Affiliations:** Department of Vascular Medicine, Room F4-253, Academic Medical Center, University of Amsterdam, Meibergdreef 9, 1105 AZ Amsterdam, the Netherlands; Department of Internal Medicine, Academic Medical Center, University of Amsterdam, Amsterdam, the Netherlands; Department of Neurology, Cincinnati Children’s Hospital, 3333 Burnet Ave, Cincinnati, Ohio USA; Department of Social Medicine, Academic Medical Center, University of Amsterdam, Amsterdam, the Netherlands

**Keywords:** creatine kinase, beta-guanidinopropionic acid, creatine, tolerability, blood pressure

## Abstract

**Background:**

Despite adequate treatment, up to 30% of treated antihypertensive patients with primary, uncomplicated hypertension remain uncontrolled. We proposed that high intracellular activity of the ATP regenerating enzyme creatine kinase (CK) increases pressor responses and hypertension risk. In line with this, we found that plasma CK activity after rest, a surrogate measure of tissue activity, is the main predictor of blood pressure levels and failure of antihypertensive therapy in the general population. In addition, the creatine analog and competitive oral creatine kinase inhibitor beta-guanidinopropionic acid effectively and safely reduced blood pressure in the spontaneously hypertensive rat. However, to our knowledge there are no human data on the safety of oral supplementation with this substance. Therefore, we will assess the tolerability of beta-guanidinopropionic acid in men, compared to creatine and placebo.

**Methods/Design:**

This is a randomized, active and placebo controlled, triple blind, double dummy, single center clinical intervention trial in 24 healthy male volunteers, 18 to 50 years old, recruited in the Netherlands. The intervention consists of one week of daily oral administration of beta-guanidinopropionic acid 100 mg, creatine 5 gram, or placebo. The primary outcome is the tolerability of beta-guanidinopropionic acid as a descriptive measure, in an intent-to-treat analysis. Other outcomes include the placebo-adjusted differences with baseline in biochemical and hemodynamic parameters, including plasma markers of muscle tissue damage, urine sodium excretion, resting sitting systolic and diastolic brachial blood pressure, supine systolic and diastolic central blood pressure, pulse wave velocity and augmentation index, heart rate, cardiac contractility, cardiac output, and total peripheral resistance.

**Discussion:**

There is an unfulfilled need for new conservative options to treat resistant hypertension. This study will provide first-in-men data on creatine kinase inhibition as a potential new class of antihypertensive drugs.

**Trial registration:**

The Netherlands National Trial Register Trialregister.nl (identifier NTR 4444), registered 9 March 2014.

## Background

Blood pressure reduction may be challenging despite the availability of several classes of antihypertensive drugs [1-5]. A substantial proportion of treated hypertensive patients, up to 30% or more does not achieve blood pressure control. Risk factors for poor control include obesity, age, African ancestry, the presence of diabetes or end organ damage; but non-adherence of the patient, the white-coat effect, therapeutic inertia of the physician, or the concomitant use of blood pressure increasing drugs may also contribute. However, a subgroup of patients with uncomplicated, primary hypertension remains who are uncontrolled despite adequate use of antihypertensive drugs [1-5]. The underlying pathophysiology in these patients is thought to be refractory to currently available drugs, causing early heart disease, stroke, and early mortality. Hence, the current scientific challenge is to develop new conservative options to lower blood pressure [1-5].

We showed in a random, multi-ethnic population sample that plasma CK activity after rest, a surrogate measure of tissue CK, is the main predictor of blood pressure, with a crude increase in blood pressure of 14 mm Hg systolic and 8 mm Hg diastolic per log CK increase [6]. Although plasma and tissue CK activity were found to be higher in men, subjects of African ancestry, and obese patients [6,7], the association was independent of sex, body mass index (BMI), or ethnicity. Therefore, we proposed that high tissue CK might increase pressor responses [6].

Cytosolic CK is tightly bound in the immediate proximity of ATP-utilizing enzymes such as Na^+^/K^+^-ATPase, Ca^2+^-ATPase, and myosin ATPase. Here, ATP synthesized by CK is preferentially used to fuel highly energy-demanding processes such as sodium retention, cardiovascular contractility, as well as remodeling of arteries, promoting hypertension [6,8]. Importantly, in accord with a causal relationship, high tissue CK preceded hypertension in animal models [9,10], as was found with high plasma CK in humans [11], and inhibition of intracellular CK substantially inhibited human vascular contractility *in vitro* [12]. Furthermore, vascular CK gene expression was strongly associated with clinical blood pressure in humans [13], and high plasma CK was found to be the main predictor of failure of antihypertensive therapy in the general population [5,14]. Finally, we recently showed in a randomized control trial of 16-week-old male spontaneously hypertensive rats versus controls (n = 16), that oral CK inhibition with the competitive CK inhibitor beta-guanidinopropionic acid (GPA) 3%, added to rat chow over 4 weeks, safely reduced blood pressure. With a systolic and diastolic baseline blood pressure of respectively 191.5 (SE 4.3) and 143.1 (SE 4.1) mm Hg, GPA significantly reduced blood pressure compared to controls by 42.7 (5.5) systolic and 35.3 (4.8) mm Hg diastolic (*P* < 0.001), respectively [15]. To our knowledge, there are no human data on the safety and effects of this potential new antihypertensive agent; we will assess the tolerability of GPA in healthy volunteers.

## Methods/Design

### Test product

#### *GPA*

**Figure 1:**
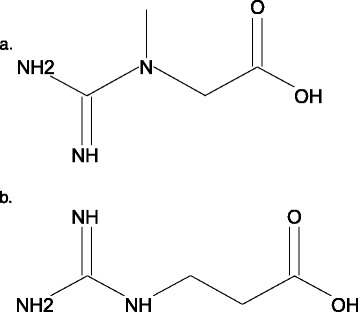
**Structural analogy between creatine and beta-guanidinopropionic acid.** Legend: Creatine **(a)** and beta-guanidinopropionic acid **(b)** have an identical molecular formula (C_4_H_9_N_3_O_2_)_,_ but creatine is methylated on its tertiary nitrogen, while in beta-guanidinopropionic acid, the methyl group is positioned in the carbon chain [16].

**Figure 2:**
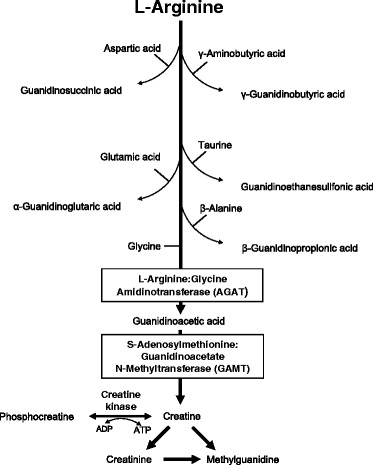
**Pathways of guanidino compound synthesis.** Legend: Guanidino compounds such as beta-guanidinopropionic acid, creatine, guanidinoacetic acid, gamma-guanidinobutyric acid, and guanidinosuccinic acid, are reported to be synthesized via transamidination of the amidino group from arginine as the major pathway, or through the urea cycle. Creatine biosynthesis involves two sequential steps catalyzed by L-arginine:glycine amidinotransferase (AGAT), and S-adenosylmethionine:guanidinoacetate N-methyltransferase (GAMT) After [17-19].

GPA or N-(aminoiminomethyl)-beta-alanine; (Cq_4_H_9_N_3_O_2_), is a structural isomer of creatine (Figure 1) [16]. GPA is generated *in vivo* via transamidination of β-alanine (Figure 2) [17-19]. The physiological concentration in human plasma is reported to range from trace amounts to 1.40 μmol/L [20,21]. Clearance is probably renal, akin to creatine, creatinine, and other guanidino compounds [17,19-21].

Despite the lack of human data on efficacy and side effects, GPA is available as a food-supplement, usually in doses of 500 mg, and is used by sportspersons to induce endurance capacity and promote weight loss [16]. GPA acts as a competitive inhibitor of cellular creatine uptake, and attenuates the flux through the cytoplasmic creatine kinase reaction [15,16,18]. CK catalyses the rapid and reversible transfer of a phosphoryl group from creatine phosphate to ADP, thereby forming creatine and ATP:

$$ ADP+ creatine\kern0.5em  phosphate\kern0.5em <=>\kern0.5em ATP+ creatine. $$

The flux through the CK reaction is linearly correlated to the concentration of creatine [22].

Nuclear genes encode four CK subunits or monomers: cytoplasmic muscle (CKM), cytoplasmic brain (CKB), ubiquitous mitochondrial (CKMT1), and sarcomeric mitochondrial (CKMT2). The enzymatic functional form can be either a homodimer (BB and MM), a heterodimer (MB), or an octamer (mitochondrial monomers), thus creating five isoenzymes. The three dimeric cytoplasmic CKMM, CKMB, and CKBB isoenzymes are predominantly expressed in striated skeletal and heart muscle (MM), heart muscle (MB); and brain and smooth muscle (BB). The two octameric mitochondrial CK isoenzymes are expressed in striated muscle and other tissue [13]. The cytoplasmic isoenzymes appear in plasma of healthy subjects, due to what is thought to be a nontraumatic proportional release from tissue entering the bloodstream through the lymphatic system [6]. Hence, plasma CK is mainly the CKMM isoenzyme.

GPA is thought to inhibit cytoplasmic, but not mitochondrial CK [15,16,18]. Although GPA is phosphorylated by cytoplasmic CK, both GPA and phosphorylated GPA are 'inefficient substrates' for the CK reaction: *in vitro* Vmax values are <1% of the Vmax values of creatine and phosphocreatine [16,18,23]. Therefore, GPA may modulate the energy status of tissues, and we speculated that this creatine analog may reduce blood pressure.

In animal studies, supplemental GPA (1 to 3%) in the diet led to skeletal muscle changes similar to the adaptations of endurance training [16]. In the unstressed heart, left ventricular systolic pressure, cardiac output, and rate of tension development were unchanged with GPA. During high workload, studies showed unchanged or reduced peak left ventricular-developed pressure and cardiac output. However, blood pressure and peripheral hemodynamic parameters were not an outcome in these studies [16]. We recently showed that feeding 16-week-old spontaneously hypertensive rats a diet containing 3% GPA reduced blood pressure [15]. Importantly, the animals appeared healthy after GPA [15,16], and the blood pressure-lowering effect was reversible after withdrawal of the analog [15].

### Manufacturing and testing

In accord with the definition for food supplements in the legislation of the European Union [24], we consider GPA as well as creatine to be food supplements, because both are naturally occurring amino-acids. GPA is a white crystalline tasteless powder, soluble in water. GPA powder is ordered at Sequoia (Sequoia Research Products, Oxford, UK). There are no reports or bans on this product or the company to our knowledge, presented on the FDA website using the FDA search engine, or online with search engine Google, as of 20 February 2014. GPA, creatine, and identical placebo capsules will be manufactured by the Pharmacy & Pharmacology Department of the Slotervaart Hospital, Amsterdam, The Netherlands. This department is GMP certificated (ISO 9001:2001). Product dossiers for GPA and creatine were written and have received formal ethical approval by the AMC Amsterdam Medical Ethics Review Committee (MERC). GPA is marketed for human use in the U.S. and Australia, but not in European countries. According to the legal guidelines of the European Union, criteria of international organs, generally accepted criteria, or national criteria are approved when a supplement is not listed in the legislation of the European Union.

Following the U.S. FDA guidelines [25], we first qualified the supplier by establishing the reliability of the supplier, with the methods mentioned above. Next, the substance was tested for purity and for cyanide compounds. Cyanide was not expected to be quantifiable [26]. However, the cyano-group in cyanamide, one of the compounds used in the formation of GPA, provides a possible source of cyanide. We established in our certified tests a purity of more than 99% (detection limit) and a cyanide level lower than 1 p.p.m. (detection limit). Cyanide occurs in many food items, with high concentrations in cassava roots, almonds, and apricot kernels, up to 7,000 mg/kg (7 parts per thousand) [27].

In Europe, Annex II of Directive 88/388/EEC on flavorings sets the following maximum levels for hydrocyanic acid in foodstuffs and beverages: 1 mg/kg in food or beverages, with the exception of 50 mg/kg in nougat, marzipan or similar products, and 5 mg/kg in canned stone fruit [28]. With 100 mg GPA containing <1 p.p.m. cyanide, the contribution to the daily intake will be <0.1 microgram/day.

### Storage and distribution

GPA and creatine capsules will be stored at room temperature. The participants will receive the test products at the hospital and will be instructed to store the capsules at room temperature at home.

The test products will be labeled with a study number, with the pharmacy holding the key to the content until the end of the data collection.

### Dose calculation

For GPA, we used the FDA guidance on Estimating the Maximum Safe Starting Dose in Initial Clinical Trials in Adult Healthy Volunteers [29]. This guidance outlines a process for deriving the maximum recommended starting dose for first-in-human clinical trials in adult healthy volunteers, and recommends a standardized process by which the maximum recommended starting dose can be selected. The purpose of this process is to ensure the safety of the human volunteers [29]. Toxicity should be avoided at the initial clinical dose. However, doses should be chosen that allow reasonably rapid attainment of phase 1 trial objectives [29]. The major elements of this process are as follows:

1. Determination of the no observed adverse effect levels (NOAELs) in the tested animal species
2. Conversion of NOAELs to human equivalent doses (HED)
3. Application of a safety factor.

### 1. No observed adverse effect level (NOAEL) determination

In animal studies, GPA was administered through the diet in concentrations of 1% or more over 8 weeks without apparent adverse effects [16]. In animals weighing 200 grams eating 20 grams per day, we calculated a 'no observed adverse effect level' of 1,000 mg/kg/day.

Furthermore, in a patent application, Meglasson *et al*. recommended a human dose of 1 to 500 mg/kg/day based on his research in mice and rhesus monkeys [30]. In this paper, rhesus monkeys weighing 9 kg were treated with oral GPA 48 mg/kg/day (432 mg per monkey per day) over 2 weeks without apparent adverse events.

### 2. Conversion of the no observed adverse effect level (NOAEL) to human equivalent dose (HED)

We converted the oral NOAELs in rats and monkeys (resp. 1,000 mg/kg/day and 48 mg/kg/day) to oral HEDs based on an algorithm proposed by the FDA based on body surface area [29]. This algorithm proposes a conversion factor from rat to human of 0.16 times the rat dose; and of monkey to men of 0.32 the monkey dose (in mg/kg/day; for a man of 60 kg) resulting in HEDs of resp. 160 mg/kg/day and 15 mg/kg/day for a man of 60 kg.

### 3. Application of a safety factor

A safety factor should be applied to the HED to increase assurance that the first dose in humans will not cause adverse effects. The use of the safety factor is based on the possibility that humans may be more sensitive to the toxic effects of a substance than predicted by the animal models, that bioavailability may vary across species, and that the models tested do not evaluate all possible human toxicities, or cannot be expressed by animals or easily measured, such as headache or nausea. We conservatively chose 15 mg/kg/day oral dose for our final calculations of the human dose, because this is the lowest dose, and because of the closer allometric relationship between monkey and man [29]. FDA advises a safety factor of at least 10. Based on an average weight of a male volunteer of 75 kg, we calculated a starting oral dose for this phase 1 study of 75*1.5 mg/day = 112.5 mg/day; we will use 100 mg/day.

### Creatine

Creatine, which has an identical molecular formula as GPA, was chosen to simultaneously assess the effect of the synergist on peripheral hemodynamics. The average daily rate of creatine synthesis in healthy omnivorous males is estimated to be 1.3 g [31]. We will use 5 g as recommended in studies on creatine supplementation. No side effects are apparent at this dose [31,32].

### Study design

We wrote the protocol using the SPIRIT '(Standard Protocol Items: Recommendations for Interventional Trials)' recommendations and the 'Template for Intervention Description and Replication (TIDieR) checklist' [33,34].

The ABC trial is a randomized, placebo and active controlled, double-dummy, participant, intervention provider, and outcome assessor (triple) blinded, parallel group, single center (Academic Hospital of the University of Amsterdam), exploratory trial, with three arms: a primary outcome of tolerability of GPA, in comparison with creatine and placebo.

Randomization will be performed by an independent party, the Clinical Pharmacy Unit of the Academic Hospital of the University of Amsterdam, using a computer-generated, nonadaptive, and restricted randomization scheme and a 1:1:1 allocation ratio. The Pharmacy will generate the random allocation sequence. All participants who give consent for participation and who fulfill the inclusion criteria will be randomized to receive GPA 100 mg and creatine placebo matching active creatine 5 gram; creatine 5 gram and GPA placebo matching GPA 100 mg; or double dummy placebo over 1 week. The participant will receive the blinded, randomized trial supplements from the pharmacy. Allocation concealment will be ensured, as the pharmacy will store the allocation list and not release the randomization code until all outcome measures have been assessed and the data bank has been closed. Thus, randomization will be conducted without any influence of the investigators, outcome assessors, or participant characteristics. After assignment to interventions, trial participants, trial staff, and the outcome assessor will remain blinded to whether the participant was given a placebo or a supplement until after all outcome data have been assessed. Data analysis will be performed unblinded. We prespecified the use of accumulating data to decide whether to stop the trial early, in case of serious or unexpected side effects. Independent monitoring visits will take place before the start of the trial, within 1 month after initiation or after inclusion 5th subject; within 2 months after initiation or after inclusion 15th subject; after last patient last visit; and after the data have been entered into the database. Data entry will be verified by two independent researchers. Budget administration is by an independent organization (AMC Research BV, Amsterdam, the Netherlands).

### Objectives

The primary objective is to assess the tolerability of one week of 100 mg oral GPA daily, as compared to placebo. Secondary objectives include the comparison of tolerability with creatine, and the effect of one week of oral GPA on hemodynamic parameters, including peripheral and central blood pressure, and cardiac contractility as compared to creatine and placebo. The tertiary objective is to assess the effect of one week of oral administration of GPA on biochemical parameters, including ADP-induced platelet aggregation [35], compared to creatine and placebo.

### Eligibility

We will include healthy men aged 18 to 50 years, with a normal, nonobese body mass (BMI 18.5 to 29.9 kg/m^2^). Exclusion criteria include high blood pressure or the use of antihypertensive drugs at baseline, (history of) cardiovascular disease including TIA and stroke; the use of plasma CK-increasing drugs including statins; use of acetylsalicylic acid or nonsteroidal anti-inflammatory drugs in the two weeks prior to the first visit; neuromuscular or endocrine disorders; vasculitis; HIV infection; infectious hepatitis; personal or family history of bleeding disorders; sickle cell anemia or other hereditary anemia; smoking; current use or use within two months prior to start of the trial of beta-guanidinopropionic acid or creatine; and abnormalities in glucose, lipid spectrum, thyroid, kidney, or liver biochemistry parameters in the plasma. To stabilize and standardize plasma CK activity during the trial, participants are instructed not to perform exercise three days prior to the baseline visit or during the intervention in the first week [6].

### Statistical analysis

#### Study outcomes

The primary outcome is the tolerability of GPA after oral administration in healthy male volunteers versus placebo as a descriptive measure, in an intent-to-treat analysis. Other outcomes are to compare the tolerability of GPA with creatine, and differences in hemodynamic and biochemical parameters between treatment arms.

#### Sample size calculation

This is a first-in-man study with GPA, with allometric data available from other species. According to the European Medicines Agency (EMEA) guidelines [36], we will include eight subjects in each arm, to assess the tolerability of GPA versus placebo and creatine over one week.

### Recruitment strategy

We will utilize two primary resources for identifying and recruiting potential subjects: advertising and identification in our Healthy Volunteer Research Database. The advertisement has prior approval of the MERC. A dedicated trial staff member will respond to inquiries about participation in the trial on the same day, using a participant information letter approved by the MERC. Screening will continue until the target population is achieved (24 subjects). The enrollment period is planned to extend over 9 months, until December 2014.

### Clinical investigation

The participants will be instructed to come to the hospital in the morning after an overnight fast for all visits during the intervention, which is the first week of the trial (Visit 1 to 5).

### Intervention

The intervention will be provided under supervision of a medical doctor. The duration of the intervention will be 7 days. To ensure intervention adherence, trial supplements are ingested by the volunteer in the presence of the trial staff during the hospital visits. In addition we will use pill counts for the supplements taken at home.

### Time line clinical studies

**Figure 3:**
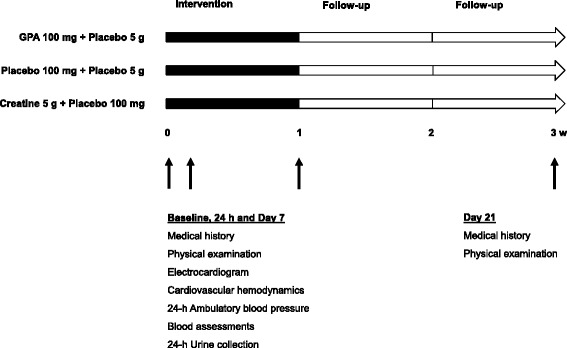
**Trial time line.** Legend: The duration of the intervention is 7 days. The trial supplements start at Day 1, after baseline measurements, inclusion, and randomization at Day 0 (baseline). After 24 h and 7 days of trial supplements, baseline measurements will be repeated, with the last visit at Day 21, to assess side effects.

The time line is depicted in Figure 3. In brief, clinical studies will be performed at baseline, and 1 day and 7 days after the trial supplements are used. The final assessment of tolerability is at Day 21.

### Questionnaire

The participants will receive a questionnaire to assess tolerability at home during the week of intervention and in the 2 weeks after the intervention. The questionnaire encompasses the perceived side effects of the trial supplements, using check boxes and free text space. The questionnaire was piloted with healthcare professionals and healthy volunteers.

### Electrocardiography and Hemodynamics

On Day 0 (baseline), 1 (in the first hour after intake trial supplements), 2 (after 1 day of trial supplements), and 8 (after 7 days of trial supplements) of the intervention period, we will measure sitting brachial systolic and diastolic blood pressure with an Omron M4 oscillometric device (Omron Healthcare Europe BV, Hoofddorp, the Netherlands) after 5 minutes of rest with an adjusted cuff size on the left arm, at heart level. In addition, we will perform electrocardiography (MAC 5000 Resting ECG System; GE Healthcare; Boston, MA) and ambulatory 24-hour blood pressure monitoring (Spacelabs 90217 Ambulatory Blood Pressure Monitor, Spacelabs Inc. Redmond, WA, USA). Furthermore, at baseline, Day 2, and Day 8, we will estimate central blood pressure, pulse wave velocity, and the augmentation index with an Arteriograph (Tensiomed Kft, Budapest, Hungary); and heart rate, cardiac contractility, cardiac output, and total peripheral resistance using a Nexfin BMEYE, (Amsterdam, the Netherlands) blood-pressure monitor for continuous noninvasive finger arterial blood pressure measurement.

### Laboratory studies

All laboratory studies are after an overnight fast. At baseline, we will assess plasma GPA, resting plasma CK (after 3 days without heavy exercise), glucose, insulin, lipid profile, creatine, creatinine, liver enzymes (ASAT, ALAT, gamma GT), LDH, cardiac troponin, myoglobin, TSH (to exclude subclinical hypothyroidism associated with high plasma CK), sodium, potassium, platelet count, coagulation tests (aPTT, PT), and ADP-induced platelet aggregation (area under curve at final concentration ADP 0.1, 0.2, 0.5, 1, and 2 μmol/L). Furthermore, in collected 24-h urine we will assess GPA, creatine, creatinine, urea, sodium, and potassium. Tests will be repeated after 1 day and after 7 days of trial supplements, with the exception of TSH, aPTT, and PT. ADP-induced platelet aggregation will repeated at Day 8 only.

### Concomitant care and interventions

There is no relevant concomitant care and no other interventions are permitted during this trial of healthy volunteers.

### Safety

The investigator will inform the subjects and the MERC if any event occurs, on the basis of which it appears that the disadvantages of participation may be significantly greater than was foreseen in the research proposal. The study will be suspended pending further review by the MERC, except insofar as suspension would jeopardize the subjects’ health. The investigator will take care that all subjects are kept informed.

### Adverse and serious adverse events

We do not expect any adverse effects from this low dose study. There are no FDA or other reports in formal or informal sources on the side effects of ingestion of this dose of GPA or creatine in animals or humans. Therefore, a 1-day first-in-men study that we had proposed, with hourly observation and physical and laboratory examination of the subjects, was deemed unnecessary by the MERC. Subjects with hypertension, a history of cardiovascular, liver, or kidney disease, or with laboratory abnormalities at baseline will be excluded. Adverse events reported by the included subject spontaneously or through the questionnaire, or observed by the trial staff or health care worker, will be recorded and judged by the study group and the independent physician. The trial staff will convert reported symptoms to a standard lexicon, the Common Terminology Criteria for Adverse Events (CTCAE) [37], to facilitate international scientific reporting. Adverse effects will be classified based on the FDA guideline [29], as overt toxicity (for example, clinical signs, macro- and microscopic lesions); surrogate markers of toxicity (for example, plasma liver enzyme levels); or other adverse effects. The ADR probability scale [38] will be used to assess the causal relationship between trial supplement use and any reported adverse event. Adverse events will be followed until they have abated, or until a stable situation has been reached. Depending on the event, follow-up may require additional tests or medical procedures.

### Retention and withdrawal of individual subjects

We will actively monitor retention, and once enrolled, we will make every reasonable effort to follow the participant for the entire study period. In the trial design, we put great effort into limiting the participant’s burden related to visits and procedures, including the calculation of an appropriate NOAEL and HED, and the predominant use of noninvasive assessments. Participants will receive a financial compensation within the MERC guidelines. However, subjects can leave the study at any time for any reason without any consequences. Participants will be given the option to be followed up on certain outcome measures only, if this would lead to retention. Nonadherence will in itself not be a reason to exclude the participant. We will collect the reasons for nonadherence and nonretention where possible. In addition, the investigator can decide to withdraw a subject from the study for any medical reasons. Upon withdrawal, subjects will be replaced.

### Emergency unblinding

To ensure the overall quality of the trial, code breaks will occur only when knowledge of the actual supplement given is absolutely essential for further management of the participant. This will be decided by the independent physician.

### Premature termination of the study

If, despite our expectations, any serious side effect is observed in the volunteers, the study may be stopped prematurely.

### Quality control

We will ensure that quality controls will be executed throughout the conduct of the study, with regard to participant selection, data collection, data processing and reporting. Additionally, trial staff who collect the data will be well-trained according to standard operating procedures, in the study requirements, use of the questionnaire, counseling for adherence, standardized measurement of height, weight, brachial blood pressure, electrocardiography and noninvasive hemodynamics, as well as for requirements for laboratory specimen collection including morning urine samples. On every day of the data collection, we will monitor and ascertain the performance of our measurement devices. Our database was designed to allow checks on the completeness of the entered data and basic data checks, and we will use independent double data-entry followed by matching and checking for data-entry errors. Data cleaning will be performed according to expert consensus. Finally, we will check internal and external consistency of the analyzed data before writing reports.

### Ethical considerations

The study will be conducted according to the principles of the Declaration of Helsinki (Adopted by the 18th WMA General Assembly, Helsinki, Finland, June 1964, and amended by the 59th WMA General Assembly, Seoul, October 2008), and in accordance with the Dutch Medical Research Involving Human Subjects Act (WMO). Prior to undertaking any study related procedures, each participant will receive a verbal and written explanation of study aims, methods, and potential side effects. The participants will provide written informed consent. The full study protocol is approved by the AMC Amsterdam Medical Ethics Review Committee on 25 November 2013 (MERC reference number 38368.018.12). All study-related information will be stored securely at the study site and participants’ personal study information will not be released without his written permission.

### Handling and storage of data and documents

Handling of personal data will comply with the Dutch Personal Data Protection Act. Data will be entered anonymously in a database designed for the study, with a code of which the key will be held by the clinical project leader (LMB). The original study forms, extracted data, and biological samples will be kept at the hospital for 15 years, with access restricted to the trial staff.

## Discussion

Hypertension is still the main risk factor for premature death [1]. Despite the ample availability of antihypertensive drugs and the adequate use whereof, it is estimated that around 10 to 30% of the hypertensive patients are not controlled with currently available drug regimens. Currently, there is an unfulfilled need for new conservative options to treat resistant hypertension [1-5].

This study is based on incremental data indicating that the ATP regenerating enzyme creatine kinase enhances the energy demanding processes involved in hypertension, including vascular contractility and salt retention, and that the creatine analog and competitive CK inhibitor GPA reduces blood pressure in animal studies [5,6,8-16]. Hence, this is a first-in-men study of what might become a new class of antihypertensive drugs. In this study, we will collect data with close adherence to the US FDA and European guidelines. We expect no difference in reported adverse effects between GPA, creatine, and placebo. This study will increase the knowledge on the effect of moderate reversible cytoplasmic creatine kinase inhibition on the human cardiovascular system and provide data on tolerability and hemodynamic parameters. Beta-guanidinopropionic acid doses are low, aimed at preventing toxicity in this first-in-men study. This limits the study of the efficacy of the drug.

## Trial status

The trial is currently recruiting participants.

## Abbreviations

AGAT: L-arginine:glycine amidinotransferase
BMI: body mass index
CK: creatine kinase
CKB: cytoplasmic brain CK
CKM: cytoplasmic muscle CK
CKMT1: ubiquitous mitochondrial CK
CKMT2: sarcomeric mitochondrial CK
CTCAE: Common Terminology Criteria for Adverse Events
EMEA: European Medicines Agency
GAMT: S-adenosylmethionine:guanidinoacetate N-methyltransferase
GPA: beta-guanidinopropionic acid
HED: human equivalent doses
MERC: Medical Ethics Review Committee
NOAEL: no observed adverse effect level
SPIRIT: Standard Protocol Items: Recommendations for Interventional Trials
TIDieR: Template for Intervention Description and Replication
